# A task-driven cerebral angiographic imaging based on CT perfusion

**DOI:** 10.3389/fneur.2023.1328184

**Published:** 2024-01-18

**Authors:** Cheng Wang, Siqi Chen, Donghua Mi

**Affiliations:** ^1^Research Center for Medical Artificial Intelligence, Shenzhen Institutes of Advanced Technology, Chinese Academy of Sciences, Shenzhen, China; ^2^Department of Neurology, Beijing Tiantan Hospital, Capital Medical University, Beijing, China

**Keywords:** stroke, cerebrovascular outflow, vessel centerline, arteriography, venography, vascular straightening

## Abstract

**Introduction:**

Current clinical computed tomography arteriography (cCTA) and clinical computed tomography venography (cCTV) images often display restricted cerebrovascular profiles, incomplete brain tissue segmentation, and incomplete artery-vein segmentation. Especially for vessels associated with diseases, capturing their complete profiles proves challenging.

**Methods:**

In this work, we developed a Task-driven Cerebral Angiographic Imaging (TDCAI) technique using computed tomography perfusion (CTP) images of stroke patients. A evaluation on intracranial hemorrhagic stroke (IHS) and acute ischemic stroke (AIS) cases was performed with CT perfusion imaging. The TDCAI technique processed the CTP images, resulting in supplementary diagnostic images, including CTA, CTV, centerline images of the vessels-of-interest [internal carotid artery (ICA) for AIS patients, Labbé vein for IHS patients], and straightened images of the vessels-of-interest.

**Results:**

We conducted a comparison between the obtained CTA/CTV images and the cCTA/cCTV images in terms of overall image quality and visibility of the vessels-of-interest. By constructing a virtual vascular phantom, we extracted its centerline and compared it with the actual centerline to calculate maximum and average deviations. This allowed us to evaluate both the accuracy of the centerline extraction algorithm and its capability to resist the influence of side branches. We assessed whether vascular stenosis and dilatation could be expressed in straightened vessel images, conducting statistical analyses to establish the superiority of TDCAI technique.

**Discussion:**

This study proposes a TDCAI technique to eliminate bone and soft tissue interference, effectively segregate the comprehensive cerebral venous and arterial systems, and extract centerlines and straighten the vessels-of-interest, which would aid doctors in assessing the outflow profiles of vessels after a stroke and seeking imaging biomarkers correlated with clinical outcomes.

## 1 Introduction

Acute Ischemic Stroke (AIS) is a prevalent medical condition characterized by the sudden loss of blood circulation to a part of the brain, leading to a myriad of neurological complications (1). The likelihood of experiencing AIS rises significantly with age, making it a common concern in the elderly population (2). This condition is becoming more widespread globally, a trend primarily attributed to modifiable risk factors (3). Particularly in developing countries, lifestyle choices, such as poor diet and lack of physical activity, contribute to the increasing incidence of AIS. Interestingly, AIS does not affect all demographics equally (4). Women, in particular, face a higher risk of AIS, with the vulnerability starting to rise during the perimenopausal period and persisting into older age groups. Hormonal changes and other gender-specific factors might play a role in this predisposition (5, 6).

Intracranial hemorrhagic stroke (IHS) is one of the most severe category of acute stroke, leading to high mortality and morbidity (7). About one-third of patients with an IHS die within 1 month after stroke onset and the remaining patients may suffer from a variety of functional disabilities, a higher risk of IHS recurrence and complications with other neurological diseases (8). It is readily apparent that the diagnosis and individualize treatment decision of patients with IHS are crucial components of the emergency workup.

In AIS and IHS, taking into account the overall cerebral circulation involving both arterial and venous outflow levels is crucial for better treatment decision-making (9, 10). In contemporary clinical settings, doctors observe cerebral arteries using computed tomography arteriography (cCTA) and cerebral veins through clinical computed tomography venography (cCTV) images (11–13). Nevertheless, these methods often show limited cerebral vascular profiles, inadequate brain tissue segmentation, and incomplete artery-vein separation. Particularly, vessels associated with diseases might be entirely invisible. The purpose of this work is to simultaneously address these technical limitations while ensuring applicability across various diagnostic tasks. This includes observing cerebral arteries and ICA for AIS patients, as well as examining cerebral veins and Labbe veins for IHS patients. In this work, we developed a CT perfusion-based Task-driven Cerebral Angiographic Imaging (TDCAI) technique that can provide high-quality CTA and CTV images. Additionally, it also provides centerline images and straightened images of the vessels-of-interest. As a result, TDCAI has the potential to aid clinicians in making more informed treatment decisions.

## 2 Materials and methods

### 2.1 Subjects

We retrospectively collected CTP image datasets and clinical information from patients diagnosed with spontaneous intracranial hemorrhagic stroke (IHS) (10 patients) or acute ischemic stroke (AIS) (10 patients) who were admitted to the emergency room of Beijing Tiantan Hospital. According to the hospital's stroke management protocol, suspected stroke patients underwent a non-contrast CT (NCCT) scan upon admission to determine the stroke type and subtype.

### 2.2 The proposed TDCAI technique

**Figure 1:**
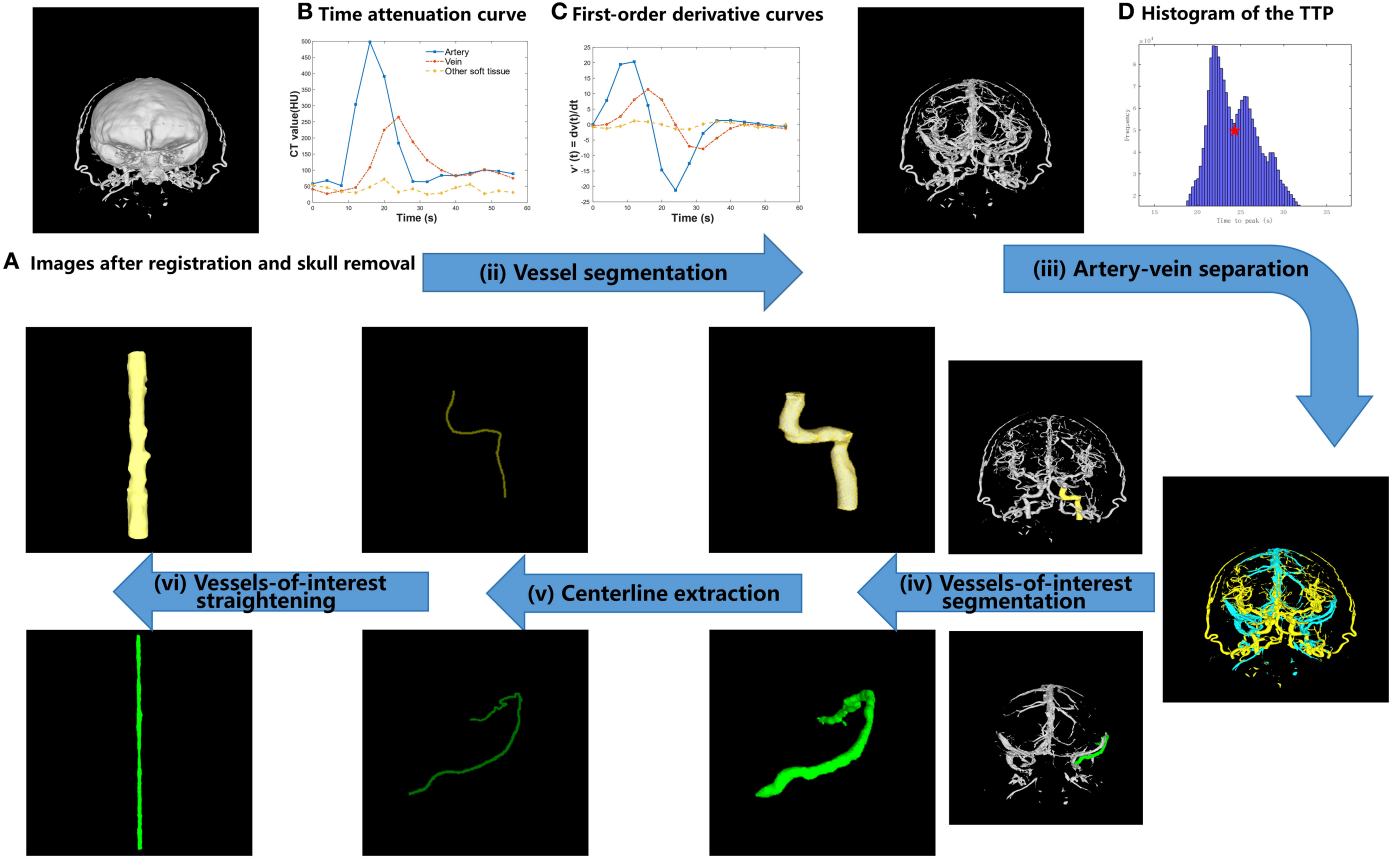
Proposed TDCAI workflow. **(A)** Images after registration and skull removal. **(B)** Time attenuation curve. **(C)** First-order derivative curve. **(D)** Histogram of the TTP.

The proposed TDCAI technique's workflow is depicted in Figure 1 and encompasses four primary stages: (i) registration and skull removal, (ii) vessel segmentation, (iii) artery-vein separation, (iv) vessels-of-interest segmentation, (v) centerline extraction, and (vi) vessels-of-interest straightening.

#### 2.2.1 Registration and skull removal

ImageJ image registration software toolkit (Fijiyama) (14, 15) was used to perform the rigid image registration with six degrees of freedom for the CTP images. Using the skull at the first time frame as the benchmark, image volumes acquired at the following time frames were registered to the image volume acquired at the first time frame.

A skull mask was obtained by preserving any voxels with an intensity value ≥155 HU (16). The skull mask was applied to the registered CTP image volumes to remove skulls and extracranial anatomical structures.

#### 2.2.2 Vessel segmentation

The temporal variation of the linear attenuation coefficients with respect to a given image voxel was referred to as the time attenuation curve (TAC) at the given voxel (17). Different types of tissues, including arteries, veins, and other brain tissues (referred to as Other soft tissues), in images (Figure 1B) show different types of temporal variations. Therefore, it is feasible to separate these types of tissues according to their different behaviors of temporal variations.

The first-order derivatives of the Gaussian function were analytically calculated and then digitized as two filters with a kernel size of 1 × 5 and parameters σ = 3. As shown in Figure 1C, the first-order derivative curves were generated via convolving the TAC with the corresponding filters. As shown in Equation (1), the absolute values of the first-order derivative curves were summed up and the summed values were referred to as *FG* in the equations. In CT images of most patients, using a thresholding value, other brain tissues could be effectively removed.


(1) $$\begin{array}{ll} FG & =\overset{T}{\underset{i=1}{\sum }}|v_{i}^{{\;}^{\prime }}| \end{array}$$

where, *i* denotes the time index of the digitized first-order derivative curves, $v_{i}^{{\;}^{\prime }}$, *T* denotes the number of time frames.

#### 2.2.3 Artery-vein separation

The time when the TAC of each voxel reaches its peak (time to peak, TTP in short) was calculated and referred to as using the following equations (18).


(2) $$\begin{array}{ll} a & =\frac{v_{I+1}^{\prime }-v_{I}^{\prime }}{t_{I+1}-t_{I}} \end{array}$$

(3) $$\begin{array}{ll} b & =v_{I+1}^{\prime }-at_{I+1} \end{array}$$

(4) $$\begin{array}{ll} t_{ttp} & =-\frac{b}{a} \end{array}$$

where, *t*_*I*+1_ represents the time when $v_{I+1}^{\prime }<0$ occurs at the first time, and *t*_*I*_ represents the nearest sampled time earlier than *t*_*I*+1_.

A histogram was drawn by counting the TTP values of all image voxels, as shown in Figure 1D. The TTP value of the lowest valley between the two peaks (denoted by the red five-pointed star in Figure 1D) was selected as the threshold value for artery-vein separation and denoted as *V*_*ttp*_. *V*_*ttp*_ was used to separate arteries from veins based on the prior knowledge that the TTP value of the contrast agent arriving at arteries is statistically earlier than that at the veins (19, 20).

#### 2.2.4 Segmentation of vessels-of-interest

We hope to observe ICA (represented by the yellow color in Figure 1) on the AIS lesion side and Labbe (represented by the green color in Figure 1) on the IHS lesion side. We used the software toolkit (3D Slicer) (21) to manually delineate vessels-of-interest.

#### 2.2.5 Centerline extraction

A centerline can be defined as a line drawn within the vessel-of-interest, consisting of the spherical centers of the largest inscribed spheres on the vessel wall of the vessel-of-interest (22, 23). First, doctors set the starting and ending points of the centerline for the vessel-of-interest through a human-computer interaction interface. Next, the outer contour image of the vessel-of-interest, known as the vessel wall image, is extracted. Then, a three-dimensional mesh is generated from the vessel wall image, and centroids of the cross-sections of the three-dimensional mesh are computed. The Dijkstra algorithm is employed to determine the shortest distance between the starting and ending points among all centroids. The line connecting all centroids within this distance becomes the vessel centerline.

#### 2.2.6 Vessel-of-interest straightening

**Figure 2:**
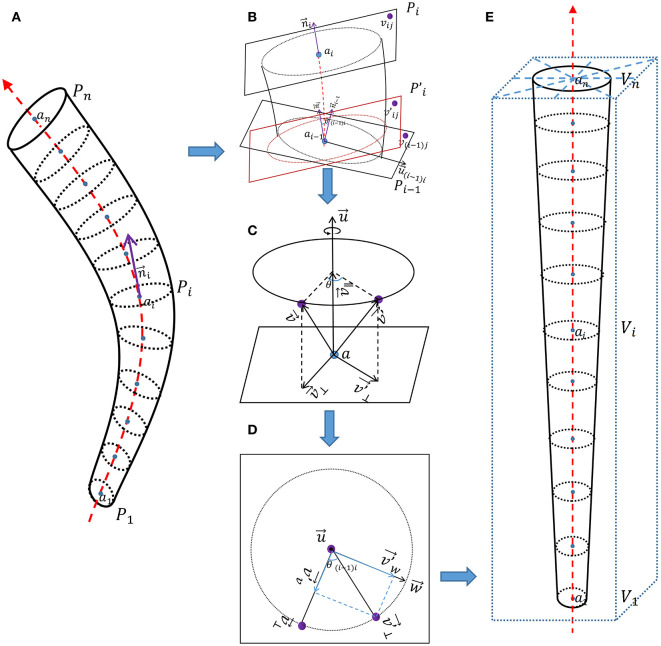
Schematic diagram of vessel straightening. **(A)** Vascular phantom. **(B)** Detailed diagram of adjacent two normal planes. **(C)** Detailed diagram of the rotation of corresponding points. **(D)** Detailed diagram of vector decomposition during the rotation process of corresponding points. **(E)** Straightened vascular phantom.

As shown in Figure 2, according to the three-dimensional coordinates of vessel centerline, interpolation and multi-plane reconstruction were performed in the four-dimensional matrix of vessels of interest to obtain the four-dimensional matrix of straightened vessel of interest.

As shown in Figure 2A, {*a*_1_, *a*_2_, …, *a*_*n*_} represent coordinate of points on the vessel centerline, {*P*_1_, *P*_2_, …, *P*_*n*_} represent coordinates on each normal plane on the vessel centerline, *P*_*i*_ is the set of three-dimensional coordinates (*v*_*ij*_) of all points on the i-th normal plane along the vessel centerline.


(5) $$\begin{array}{l} P_{i}=\left\{v_{ij}|1\leq j\leq m\right\} \end{array}$$

${\overset{\to }{n}}_{i}$ represent tangent vector of points on the vessel centerline, and it is also the normal vector of the normal plane *P*_*i*_.


(6) $$\begin{array}{l} \begin{array}{c} {\vec{n}}_{i}=\{\begin{array}{c} {\vec{a}}_{2}-{\vec{a}}_{1},\;\;i=1\\ {\vec{a}}_{i+1}-{\vec{a}}_{i-1},\;\;i\in \left(1,n\right)\\ {\vec{a}}_{n}-{\vec{a}}_{n-1},\;\;i=n \end{array} \end{array} \end{array}$$

As shown in Figure 2B, for the two adjacent normal planes *P*_*i*−1_ and *P*_*i*_ on the vessel centerline. *P*_*i*_ can be seen as the plane ${P^{\prime }}_{i}$ obtained by first rotating *P*_*i*−1_ is then translated by plane ${P^{\prime }}_{i}$ to obtain *P*_*i*_. θ_(*i*−1)*i*_ is the included angle between two adjacent tangent lines ${\overset{\to }{n}}_{i}$ and ${\overset{\to }{n}}_{i-1}$.


(7) $$\begin{array}{l} \theta_{\left(i-1\right)i}=arccos\left(\frac{{\overset{\to }{n}}_{i}\cdot {\overset{\to }{n}}_{i-1}}{\left|{\overset{\to }{n}}_{i}||{\overset{\to }{n}}_{i-1}\right|}\right) \end{array}$$

${\overset{\to }{u}}_{\left(i-1\right)i}$ represent rotational axis between two adjacent tangent vectors. For ease of calculation, we set ${\overset{\to }{u}}_{\left(i-1\right)i}$ as an unit vector;


(8) $$\begin{array}{l} {\overset{\to }{u}}_{\left(i-1\right)i}=\frac{{\overset{\to }{n}}_{i}\times {\overset{\to }{n}}_{i-1}}{∥{\overset{\to }{n}}_{i}\times {\overset{\to }{n}}_{i-1}∥} \end{array}$$

We use the rotation of vectors in three-dimensional space to represent the rotation of corresponding points. As shown in Figure 2C, $\overset{\to }{v}$ rotates θ degrees along the rotation axis $\overset{\to }{u}$ passing through point a to obtain $\overset{\to }{v^{\prime }}$. During this rotation process, the rotation axis $\overset{\to }{u}$ is a quantity with both size and direction, but its size (length) is not important here. In order to eliminate the redundant degree of freedom of the length of the rotating axis $\overset{\to }{u}$, and for the convenience of calculation, we specify that the length of the rotating axis $\overset{\to }{u}$ is 1.

We decompose $\overset{\to }{v}$ and $\overset{\to }{v^{\prime }}$ into two sub vectors (${\overset{\to }{v}}_{∥}$, ${\overset{\to }{v}}_{⊥}$ and ${\overset{\to }{v}}_{∥}$, ${\overset{\to }{v}}_{⊥}$), one parallel to the rotation axis $\overset{\to }{u}$ and the other perpendicular (orthogonal) to the rotation axis $\overset{\to }{u}$.

It can be observed that ${{\overset{\to }{v}}_{∥}=\overset{\to }{v^{\prime }}}_{∥}$, ${\overset{\to }{v}}_{∥}$ is the orthogonal projection of $\overset{\to }{v}$ on the rotation axis $\overset{\to }{u}$.


(9) $$\begin{array}{l} \begin{array}{c} {\overset{\to }{v}}_{∥}=\frac{\overset{\to }{u}\cdot \overset{\to }{v}}{\overset{\to }{u}\cdot \overset{\to }{u}}\overset{\to }{u}=\frac{\overset{\to }{u}\cdot \overset{\to }{v}}{||\overset{\to }{u}|{|}^{2}}\overset{\to }{u}=\left(\overset{\to }{u}\cdot \overset{\to }{v}\right)\overset{\to }{u} \end{array} \end{array}$$

Because ${\overset{\to }{v}}_{⊥}$ and ${\overset{\to }{v^{\prime }}}_{⊥}$ are both perpendicular (orthogonal) to the rotation axis $\overset{\to }{u}$, we can consider ${\overset{\to }{v}}_{⊥}$ rotating around the rotation axis $\overset{\to }{u}$ to obtain ${\overset{\to }{v^{\prime }}}_{⊥}$ as a rotation in the same plane. Because rotation does not change the length of vectors, the trajectory of rotation can be represented by a circle. As shown in Figure 2D, we construct a vector $\overset{\to }{w}$ that is both perpendicular (orthogonal) to the rotation axes $\overset{\to }{u}$ and ${\overset{\to }{v}}_{⊥}$.


(10) $$\begin{array}{l} \left||\overset{\to }{w}||=||\overset{\to }{u}\times {\overset{\to }{v}}_{⊥}||=||\overset{\to }{u}||\cdot ||{\overset{\to }{v}}_{⊥}||\cdot \sin\frac{\pi }{2}\;=||{\overset{\to }{v}}_{⊥}|\right| \end{array}$$

The length of $\overset{\to }{w}$ and ${\overset{\to }{v}}_{⊥}$ is equal, indicating that $\overset{\to }{w}$ is also on the circle. We decompose ${\overset{\to }{v^{\prime }}}_{⊥}$ into two sub vectors (${\overset{\to }{v^{\prime }}}_{v}$ and ${\overset{\to }{v^{\prime }}}_{w}$), one perpendicular (orthogonal) to ${\overset{\to }{v}}_{⊥}$ and the other perpendicular (orthogonal) to $\overset{\to }{w}$.


(11) $$\begin{array}{l} \vec{v^{{\;}^{\prime }}}=\to {\vec{v^{{\;}^{\prime }}}}_{∥}+\;{\vec{v^{{\;}^{\prime }}}}_{⊥}=\cos\theta \vec{v}+(1-\cos\theta )(\vec{u}\cdot \vec{v})\vec{u}\\ \;+\sin\theta \left(\vec{u}\times \vec{v}\right) \end{array}$$

The process of obtaining plane ${P^{\prime }}_{i}$ by rotating plane *P*_*i*−1_ can be seen as all vectors ${\overset{\to }{v}}_{\left(i-1\right)j}$ on plane *P*_*i*−1_ rotating θ_(*i*−1)*i*_ degrees along the rotation axis ${\overset{\to }{u}}_{\left(i-1\right)i}$ (unit vector) of point *a*_*i*−1_ on the vessel centerline passing through plane *P*_*i*−1_, resulting in all corresponding vectors ${\overset{\to }{v^{\prime }}}_{ij}$ on plane ${P^{\prime }}_{i}$.


(12) $$\begin{array}{l} \begin{array}{c} v_{ij}=\cos\theta_{(i-1)i}\left(v_{(i-1)j}-a_{i-1}\right)\\ +(1-\cos\theta_{(i-1)i})\left[{\vec{u}}_{(i-1)i}\cdot \left(v_{(i-1)j}-a_{i-1}\right)\right]{\vec{u}}_{(i-1)i}\\ +\sin\theta_{(i-1)i}\left[{\vec{u}}_{(i-1)i}\times \left(v_{(i-1)j}-a_{i-1}\right)\right]+a_{i} \end{array} \end{array}$$

We know the relationship between the two normal planes *P*_*i*−1_ and *P*_*i*_, and the three-dimensional coordinates of each point on the normal plane *P*_*i*_ can be obtained using the equation based on the three-dimensional coordinates of each point on the normal plane *P*_*i*−1_.

We set *P*_0_ as a square plane perpendicular to the z-axis, with center point $a_{0}=\sqrt{m},\sqrt{m},1$ and normal vector ${\overset{\to }{n}}_{0}=\left[0,0,1\right]$, and the coordinates of each point {*v*_01_, *v*_02_, …, *v*_0*m*_} on it are known. According to the equation, we can obtain all normal plane coordinates on the vessel centerline.

Most calculated coordinates in P were not integers. Through interpolation calculation, *P* was introduced into the four-dimensional vessel image of interest to obtain the CT value (denoted as *V*) of corresponding straightened vessel.

As shown in Figure 2E, *V* was brought into the new rectangular coordinate system and arranged in order on the *z*-axis to obtain the straightened vessel image of interest.

## 3 Results

### 3.1 Comparisons of TDCAI-CTV and cCTV

**Figure 3:**
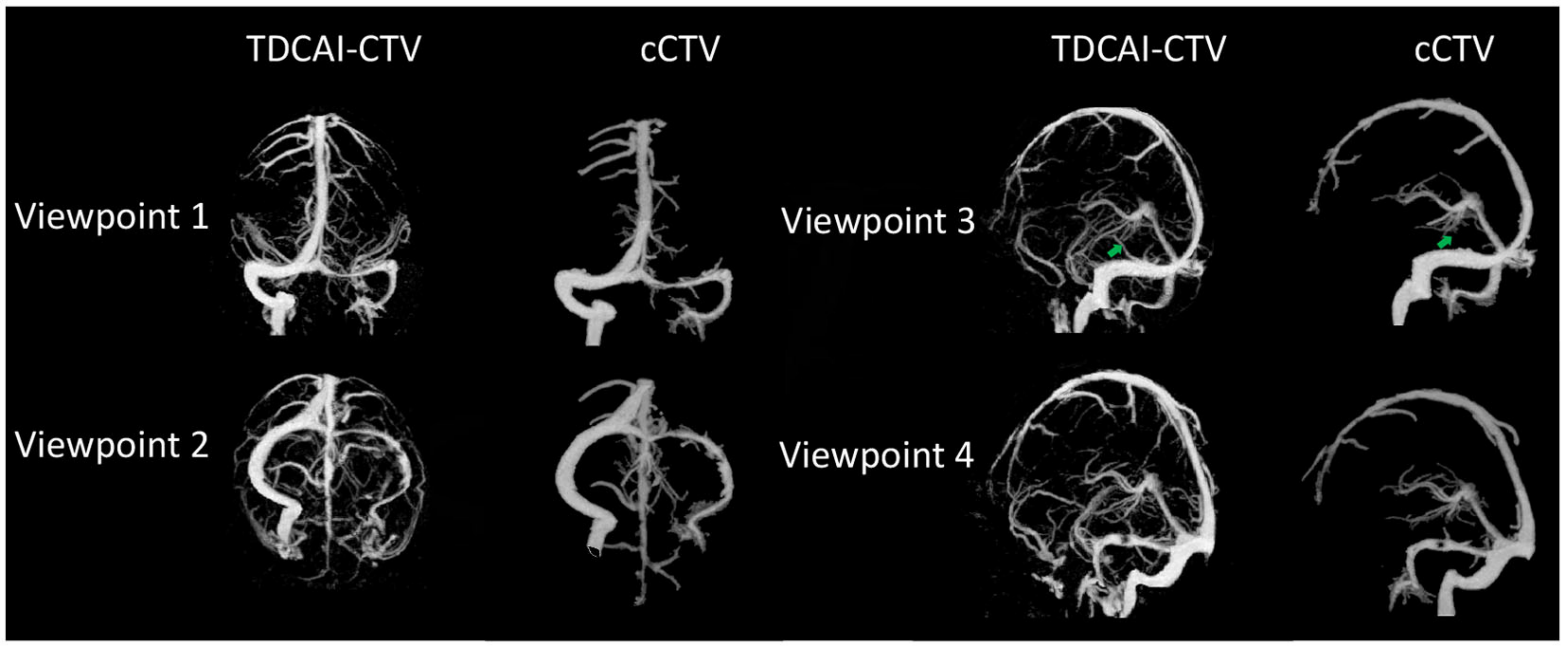
Comparisons of TDCAI-CTV and cCTV images using human subject studies.

As shown in Figure 3, the TDCAI-CTA exhibits a more detailed arterial structure, effectively visualizing the small terminal branches of arteries. Additionally, the TDCAI-CTV displays a more extensive venous structure. Significantly, the Labbé vein (indicated by the green arrow), which is linked to IHS and was previously unobservable in cCTV, is clearly discernible in TDCAI-CTV.

### 3.2 Verification of centerline extraction algorithm

**Figure 4:**
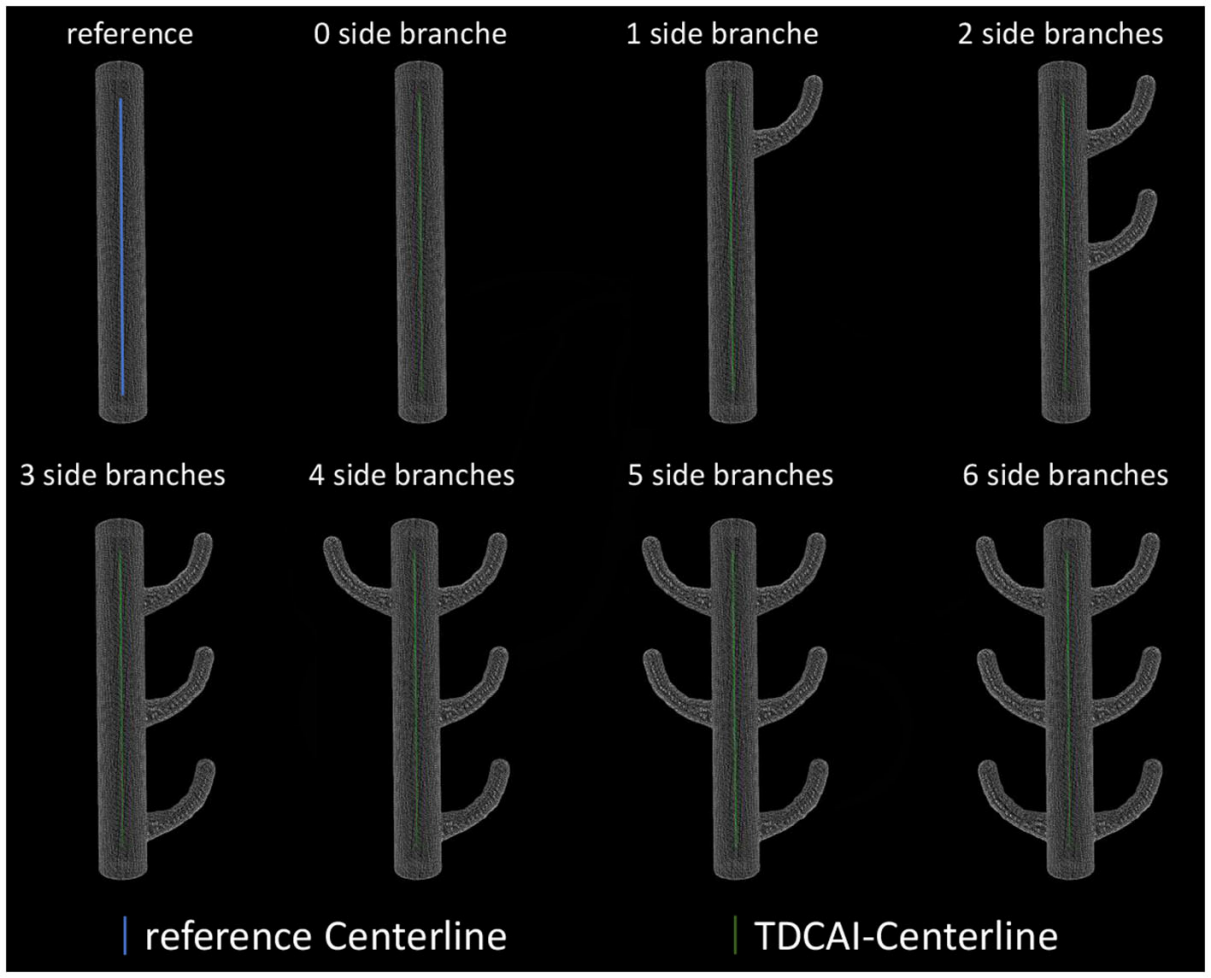
Extracting the centerline of virtual vascular phantoms.

As shown in Figure 4, we established some virtual vascular phantoms to assess the accuracy of the centerline extraction algorithm and its resistance to branching effects. The centerline was extracted and compared with the referenced centerline to compute maximum and average deviations.

The average deviation of the obtained centerlines from the referenced centerline was 0.11± 0.14 mm (maximum value appears in three side branches), with the maximum deviation being 0.36 mm (maximum value appears in six side branches).

### 3.3 Verification of the straightening effect of vessels-of-interest

**Figure 5:**
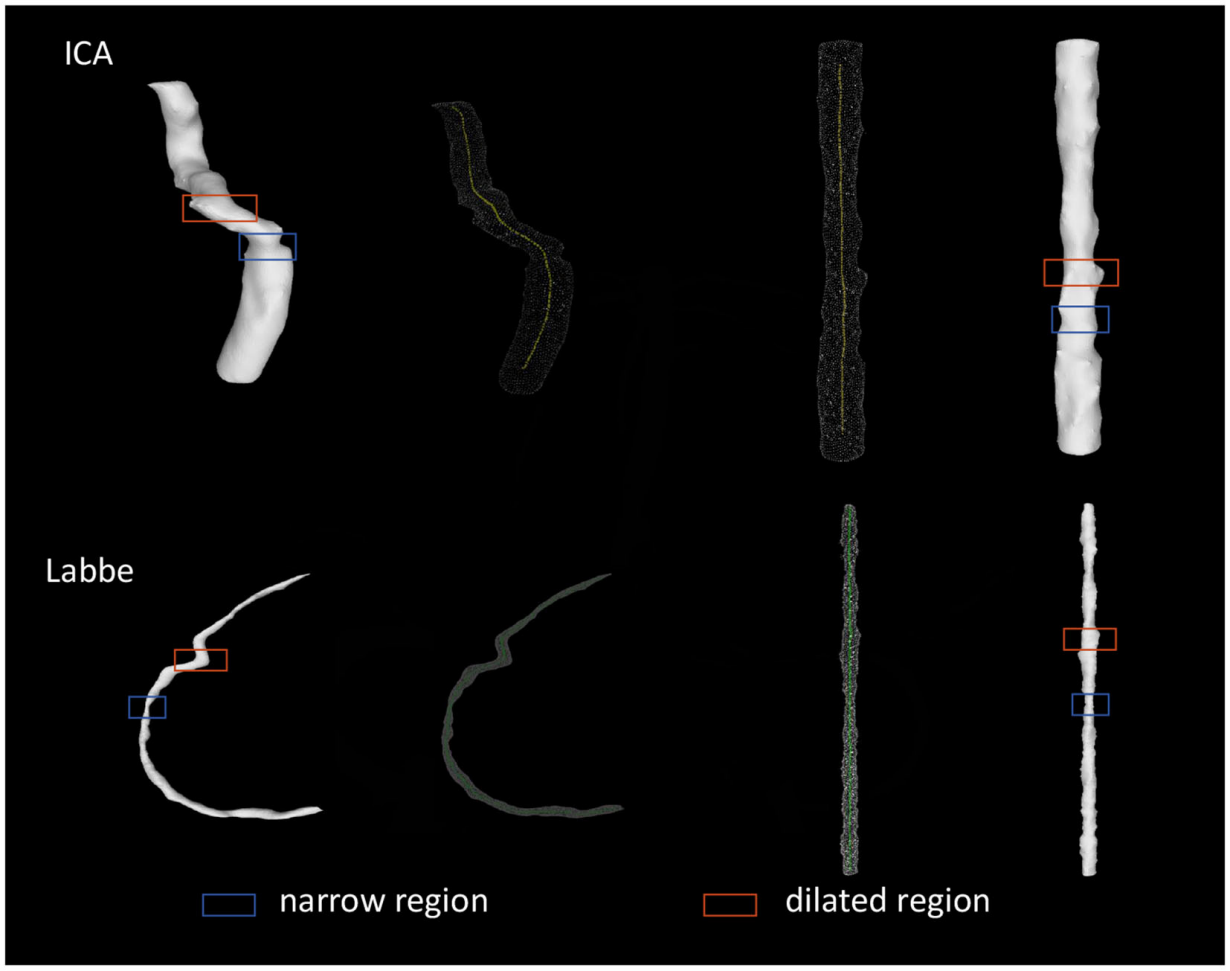
TDCAI processing of vessels-of-interest.

As shown in Figure 5, all obtained straightened images of ICA and Labbé vein were evaluated. The assessment aimed to determine the effective representation of narrow and dilated regions. Following this evaluation, a 100% accuracy rate was calculated.

## 4 Discussion

In this study, we introduced the Task-Driven Cerebral Angiography Imaging (TDCAI) technique, capable of producing high-quality arterial images, venous images, vessel centerline images, and straightened images of the vessels-of-interest. Our findings highlight TDCAI's superiority over existing clinical techniques like CTV and CTA in removing residual tissue, accurately segmenting vessels, and displaying comprehensive vascular structures.

TDCAI's enhances ability to preserve the structural details of cerebral vessels aids in identifying cerebrovascular pathologies. It provides clear visualization of cerebrovascular outflow, improving early detection of abnormal markers. It provides vessels centerline images, providing a foundation for the subsequent development of virtual endoscopes, through which doctors can observe the cerebral vascular system.

It also offers straightened images of the vessels-of-interest, enabling doctors to assess vessel conditions within a Cartesian coordinate system. This proves beneficial for potential future studies exploring the application of vessels in deep learning. Vessels in three-dimensional space often present as irregular tubes, making it challenging to extract features directly for machine learning models. When utilized as direct inputs, they tend to carry excessive and less relevant information, such as their spatial orientation, potentially complicating feature extraction. Utilizing objects in rectangular coordinates as machine learning inputs aids feature extraction, yet defining vessels themselves in this space poses difficulties. Directly using vessels in three-dimensional space as machine learning inputs without straightening them might lead the model to perceive the curvature (spatial orientation) of the vessel structure as a critical feature. However, this feature might not be relevant for our intended purpose. Our focus lies in extracting structural features and hemodynamic attributes of the vessel wall from an endoscopic perspective along the vessel's lumen. These particular features hold greater relevance to the diagnosis and prognosis of the disease. Our forthcoming research will delve deeper into these aspects, exploring new methodologies and perspectives.

Our study refrained from intervening in patient examinations and avoided bias by not selecting subjects with high-quality images, enhancing the applicability of our findings to real clinical scenarios. However, given objective variances across regions and populations, further studies are essential for validation. These efforts will play a pivotal role in advancing CT angiography imaging technology.

Our study has limitations, including a relatively small cohort. Future research should actively recruit a larger number of subjects to validate and enhance this technique. Determining factors responsible for vascular invisibility, whether innate, stroke-related, or technical, remains challenging. Practical constraints make it difficult for patients to undergo an MRI shortly after a CTP examination, making it challenging to establish a clinical reference for accurate vascular imaging in paired patients.

## 5 Conclusions

This paper proposes a TDCAI technique to remove bone and soft tissue interference, segregate cerebral venous and arterial systems, and extract centerlines while straightening vessels-of-interest. This aids doctors in assessing post-stroke vessel outflow patterns and identifying imaging biomarkers linked to clinical outcomes. Further research is required to validate and enhance the TDCAI technique.

## Data availability statement

The raw data supporting the conclusions of this article will be made available by the authors, without undue reservation.

## Ethics statement

The studies involving humans were approved by Institutional Review Board at Beijing Tiantan Hospital. The studies were conducted in accordance with the local legislation and institutional requirements. Written informed consent for participation was not required from the participants or the participants' legal guardians/next of kin in accordance with the national legislation and institutional requirements.

## Author contributions

CW: Writing–original draft. SC: Writing–review & editing. DM: Writing–review & editing.
